# Left atrial reservoir strain is an outstanding predictor of adverse cardiovascular outcomes in patients undergoing maintenance hemodialysis: Assessment via three‐dimensional speckle tracking echocardiography

**DOI:** 10.1002/clc.23815

**Published:** 2022-03-21

**Authors:** Minmin Sun, Yumeng Xing, Yao Guo, Xuesen Cao, Yuxin Nie, Xianhong Shu

**Affiliations:** ^1^ Department of Echocardiography, Shanghai Institute of Cardiovascular Diseases, Shanghai Institute of Medical Imaging, Zhongshan Hospital Fudan University Shanghai China; ^2^ Department of Nephrology, Zhongshan Hospital Fudan University Shanghai China

**Keywords:** left atrial strain, maintenance hemodialysis, speckle tracking, three‐dimensional echocardiography

## Abstract

**Background:**

There is a paucity of literature focusing left atrium (LA) in patients undergoing maintenance hemodialysis (MHD).

**Hypothesis:**

We used three‐dimensional speckle tracking echocardiography (3DSTE) to evaluate LA in MHD patients and to explore its predictive value for adverse outcomes.

**Methods:**

Echocardiography was performed on 130 consecutively enrolled MHD patients without previous cardiac diseases. Conventional and 3DSTE parameters of LA were obtained. The MHD cohort was then followed and the end point was major adverse cardiovascular events (MACEs). LA strain indices, including reservoir strain (LASr), conduit strain (LAScd), and contractile strain (LASct), were measured and compared between patients with and without MACEs.

**Results:**

Patients were prospectively followed up for a median of 40.5 (interquartile range: 26.3–48.0) months. During follow‐up, 43 patients met the end point. These patients had larger LA size and reduced LA strains (LA maximal volume indexed: 45.1 ± 11.9 vs. 33.8 ± 6.9ml/m^2^; LASr: 20.2 ± 3.5 vs. 27.2 ± 3.3%; LAScd: −12.3 ± 5.2 vs. −14.5±4.0%; LASct: −8.0 ± 4.2 vs. −13.2 ± 3.7%; all *p*<.05), compared with those without MACEs. Multivariable regression analysis showed LASr was the strongest predictor of MACEs (hazard ratio, 0.69; 95% confidence interval, 0.54–0.89; *p*=.004). Univarite Kaplan–Meier analysis revealed the incidence of MACEs in the impaired LASr (<24.2%) group was significantly higher than in the normal LASr group (log rank *p*<.001).

**Conclusions:**

LASr derived from 3DSTE is an independent predictor of MACEs and cardiac death in MHD patients, superior to LV parameters and LA volume indices.

## 1 INTRODUCTION

Cardiovascular diseases cause more than 50% of death in patients with maintenance hemodialysis (MHD).^1^ It has been suggested that left ventricular diastolic changes precede systolic dysfunction in MHD patients due to myocardial hypertrophy and interstitial fibrosis^2^ and predict cardiovascular events.^3^ Echocardiography is best suited for the assessments of cardiac performance in MHD patients because of its availability, safety, and versatility. Recently, left atrium (LA) size has increasing prognostic significance as a biomarker for diastolic dysfunction and poor prognosis in general population, elderly cohort, patients after acute myocardial infarction, and some other referral populations.^4^, ^5^, ^6^, ^7^ However, it has not been extensively studied in MHD patients for the load dependence and complicated function variability. More recently, LA strain has been used to qualify LA function and importantly, LA reservoir strain (LASr) has been shown superior than Left atrial maximal volume indexed to BSA (LAVi) in prediction of adverse cardiovascular outcomes and death.^8^ Three‐dimensional speckle tracking echocardiography (3DSTE), a new echocardiographic technology, could provide information about real‐time changes of LA during cardiac cycle. The accuracy and reproducibility of 3DSTE in evaluating LA volume and function have been previously verified compared with cardiac computed tomography^9^ and cardiac magnetic resonance.^10^

The aims of present study are (i) to investigate the LA size and function by 3DSTE to reveal LA changes in MHD patients; (ii) to explore the prognostic value of LA parameters derived from 3DSTE in the follow‐up of MHD patients.

## 2 MATERIALS AND METHODS

### 2.1 Patients

From July 2015 to August 2017, 130 MHD patients (66 male, aged from 30 to 72 years) were enrolled in this study in the Hemodialysis Unit of Zhongshan hospital affiliated to Fudan University, 82 diagnosed with chronic glomerulonephritis, 18 with IgA nephropathy, 16 with Polycystic kidney disease, and 14 with Diabetic nephropathy. They met the following criterion: regularly receiving hemodialysis three times a week for at least 6 months and having appropriate acoustic windows for echocardiography. Exclusion criteria: history of myocardial infarction, cardiomyopathy, congenital heart disease, severe valvular stenosis or regurgitation, abnormal wall motion, heart failure (LVEF < 50%), history of severe arrhythmias (see below in the definition of MACEs, diagnosed by electrocardiogram from past medical records), pulmonary hypertension caused by lung disease, moderate to large pericardial effusion.

All participants gave written informed consent and the current study was approved by the Ethics Committee of Zhongshan Hospital affiliated to Fudan University.

### 2.2 Clinical data

Each patient was physically examined and blood sample was drawn in the morning of interdialytic day: height, weight, heart rate, and blood pressure were recorded. Body surface area (BSA) was calculated as: 0.0061 × height (cm) + 0.0124 × weight (kg) − 0.0099 (m^2^). Blood urea nitrogen, serum creatinine, albumin, cholesterol, triglyceride, hemoglobin, fasting blood‐glucose, cardiac troponin T (cTnT), N‐terminal brain natriuretic propeptide (NT‐proBNP) were measured using routine methods. The medical record was reviewed and each patient was interviewed for clinical information on history of hypertension, diabetes mellitus, hypercholesterolemia, coronary heart disease, heart failure, and medical history. The amount of ultrafiltration volume was individually based on interdialytic weight gain.^11^

Recruited MHD patients received yearly evaluation and interview performed in person or by telephone. MACEs were recorded from their medical records, including cardiovascular death, newly occurred severe arrhythmias (diagnosed by electrocardiogram, including atrial fibrillation, atrial flutter, Lawn grade 3 or greater ventricular ectopy, second‐degree or greater atrioventricular block, and/or intraventricular block), ischemic events (admission due to angina and coronary revascularization) and heart failure hospitalization. The cohort was followed a median of 40.5 (interquartile range: 26.3–48.0) months and all the patients were assured to receive adequate clearance by hemodialysis.

### 2.3 Conventional echocardiography

Conventional echocardiographic images were obtained using an M5S probe (GE vivid E95, Horton). In the parasternal long‐axis view, the LA anteroposterior diameter was measured. Besides, the left ventricle (LV) end‐diastolic diameter (LVEDD), end‐systolic diameter (LVESD), interventricular septal thickness (IVST), and posterior wall thickness (PWT) were measured to calculate LV mass (LVM, g) as 1.04 × ([IVST + LVEDD + PWT]^3^ − LVEDD^3^) − 13.6. LV mass indexed to Body surface area (BSA) (LVMI) was LVM indexed to BSA. In the apical four‐chamber view, left ventricular early (E), late (A) inflow velocities, ratio between E and A velocities (E/A ratio) were measured by pulsed Doppler placing the sample volume in between the tips of the mitral valve. Pulmonary artery systolic pressure was calculated from the peak continuous‐wave Doppler velocity of the tricuspid regurgitation jet plus right atrial pressure, as assessed by the inspiratory collapse of the inferior vena cava. Pulsed‐wave tissue Doppler imaging (TDI) of the mitral annulus was used to measure myocardial velocities in peak systole (S‐lat, S‐sep), early (e'‐lat, e'‐sep), and late diastole (a'‐lat, a'‐sep), when the sample volume was placed in lateral and septal annulus of the wall. Isovolumic relaxation time and isovolumic contraction time were also acquired from TDI of the septal and lateral annulus. These measurements were acquired with a recording velocity of 100 mm/s, and the width and length of the region of interest were 1.6 and 3.1 mm, respectively. All examinations were performed according to the Recommendations for Cardiac Chamber Quantification by American Society of Echocardiography.^12^

### 2.4 3DSTE acquisition and analysis

LA three‐dimensional images were acquired with a 4V fully sampled matrix array transducer (GE vivid E95, Horton). A pyramidal full volume data set was obtained from four small real‐time subvolumes acquired from alternate cardiac cycles triggered to the *R* wave of the electrocardiogram. Sector angle and depth were regulated to include the whole left ventricle and the LA and to ensure the frame rates within 36–60 F/sec. Measurements of LA volumes and strains were then performed offline in EchoPac workstation (version 203), “4D Auto LAQ” software system. In the workstation, the 3D data was displayed in three plane views: an apical four‐chamber view; an apical two‐chamber view; and an apical LV longitudinal view. Two reference points were then set by the users in the three planes: one in the middle of the mitral valvular annulus and the other in the middle of LA roof. Then adjust the image position and angle to make the vertical line intersects the line linking MV center and the apex of the LA. Then the endocardial border was automatically traced and a mathematical model of LA was obtained. Manual adjustments were made to correct the border tracing if needed (Figure S1).

The following LA phasic volumes were calculated: LA maximal volume (LAV‐max), LA minimal volume (LAV‐min), LA pre‐systolic volume (LAV‐preA) (LA volume just before the “*p*” wave on the electrocardiogram). All volume measurements were indexed to the BSA and the following parameters representing LA phasic functions were calculated:

**Table:** 

LA phasic function	Parameters	Calculation formula
Global function	Total emptying fraction (LATEF)	(LAV‐max – LAV‐min)/LAV‐max
Reservoir function	Expansion index (LAEI)	(LAV‐max – LAV‐min)/LAV‐min
Conduit function	Passive EF (LAPEF)	(LAV‐max – LAV‐preA)/LAV‐max
Booster function	Active EF (LAAEF)	(LAV‐preA – LAV‐min)/LAV‐preA

Meanwhile, LA strain parameters were calculated during three LA phases, including reservoir strain (LASr), conduit strain (LAScd), and contractile strain (LASct)

Additionally, 3D full volume data of LV were also acquired from all subjects. LV end‐diastolic volume (LVEDV), LV ejection fraction (LVEF), and LV global longitudinal strain (LVGLS) were also analyzed off‐line in EchoPac workstation (version 203). The images acquisition and tracing proposals were performed in the same way as described in a previous literature.^13^

### 2.5 Data acquisition time

To avoid the impact of hemodialysis, echocardiography was performed on the interdialytic day in MHD cohort. To observe effects of volume load on LA, 3DSTE was also performed on 28 randomly selected patients before and immediately (within 30 min) after one hemodialysis session.

### 2.6 Reproducibility

Intra‐ and interobserver reproducibility of 3DSTE data, including LAV‐max, LAV‐min, LAV‐preA, LASr, LAScd, LASct, were assessed in 10 randomly selected patients by measuring the 3DSTE data by one observer twice on a different day and by two independent observers.

### 2.7 Statistical analysis

All data were expressed as means ± standard deviation, medians (interquartile ranges), or frequencies as appropriate. Student's unpaired *t*‐test was used to assess the difference in continuous variables with normal distributions between two groups, whereas for skewed and categorical data, Wilcoxon rank‐sum test and *χ* ^2^ tests were performed, respectively. Paired *t*‐test was used for pairwise comparison of left atrial parameters on interdialytic days, pre and post hemodialysis. Inter‐ and intra‐observer reproducibility of 3DSTE data was assessed using intra‐class correlation coefficients (ICCs). Kaplan–Meier method and Cox proportional hazard model were used to assess the association of data and MACEs. The inclusion criterion for model selection in a covariate set was predetermined as *p *< .10 in univariate Cox proportional hazard models. The covariates with *p *< .10 for predicting MACEs included LVMI, LVGLS, cTnT, LAVi (indexed LAV‐max), and LASr. Receiver operating characteristic (ROC) curves and the area under curve (AUC) were used to assess the prediction performance of LASr and other variables. DeLong tests were adopted for pairwise comparisons of efficiency. Univariate and multivariate regression analyses were adopted for predictors of LASr.

All tests were two‐tailed and a *p *< .05 was considered statistically significant. Statistical analysis was performed via IBM SPSS 16.0 software and Medcalc 14.10.2.

## 3 RESULTS

### 3.1 Characteristics of MHD patients

Six MHD patients were excluded because of poor images (defined as >2 non‐visualized segments). Clinical and biochemical Characteristics were summarized in Table S1 and conventional echocardiographic data in Table S2. The MHD cohort consisted of 124 patients on hemodialysis (64 men), with a mean age of 57 ± 12 years old. Among them, prevalence of primary hypertension, coronary heart disease, hypercholesterolemia, and diabetes was 43%, 12%, 26%, and 15%, respectively. NT‐proBNP was presented after logarithmic transformation for its extremely skewed distribution.

### 3.2 Effect of volume load on left atrial parameters

Comparisons among 3DSTE data on different time points showed LA volume parameters varied significantly. Concerning on phasic function, LA strain indices, LASr especially, showed better stability to fluid fluctuation than indices derived from volumetric methods (LASr: interdialytic vs. pre vs. post: 24.5 ± 7.6% vs. 24.4 ± 7.7% vs. 23.0 ± 8.7%, all *p *> .05) (Table S3 and Figure S2).

### 3.3 Baseline characteristics of patients with MACEs

Median follow‐up time was 40.5 (interquartile range: 26.3–48.0) months. During the follow‐up, MACEs occurred in 43 (34.7%) patients, including 8 deaths were classified as cardiac death, while 18 suffered from severe arrhythmias (diagnosed by electrocardiogram, 8 of which were atrial fibrillation, 6 atrial flutter, 3 multifocal or multiform ventricular premature beats, 1 paroxysmal ventricular tachycardia), 9 angina pectoris (diagnosed by typical symptoms and coronary artery CT scan), 5 both arrhythmia (all were atrial fibrillation) and angina pectoris, 3 myocardial infarction (diagnosed by coronary angiography).

Comparisons between Groups with and without MACEs showed patients who reached the end point were older (*p *= .01), more of whom suffered from diabetes (*p *= .034) and the blood cTnT concentration seemed higher (*p *= .063). These patients also had larger LVMI (*p *< .001) and worse LV diastolic function, shown by lower e'/a' and E/e' ratios (*p *= .027 and 0.012, respectively). 3DSTE data showed they also had significantly larger LAVi (45.1 ± 11.9 vs. 33.8 ± 6.9 ml/m^2^, *p *< .001), lower LASr (20.2 ± 3.5 vs. 27.2 ± 3.3%, *p *< .001), LAScd (−12.3 ± 5.2 vs. −14.5 ± 4.0%, *p *= .004), and LASct (−8.0 ± 4.2 vs. −13.2 ± 3.7%, *p *< .001), as well as lower LVEF (57 ± 7 vs. 61 ± 6%, *p *= .016) and LVGLS (−17.4 ± 2.5 vs. −21.2 ± 2.7%, *p *< .001) (Table 1).

**Table 1:** Comparison of 3DSTE parameters in MHD cohort grouped by MACEs

	MHD (*n *= 124)	Grouping by MACEs		
		No (*n *= 81)	Yes (*n *= 43)	*p*‐value
LA volume parameters, ml/m^2^				
LA maximal volume	38.0 ± 10.5	33.8 ± 6.9	45.1 ± 11.9	<.001*
LA minimal volume	15.2 ± 4.7	14.7 ± 4.9	16.0 ± 4.5	.227
LA pre‐systolic volume	29.3 ± 4.7	27.6 ± 8.9	32.1 ± 9.1	.038*
LA functional parameters, %				
LA total emptying fraction	60 ± 5	61 ± 4	59 ± 5	.237
LA expansion index	155 ± 29	158 ± 28	150 ± 31	.285
LA passive emptying fraction	23 ± 10	26 ± 10	19 ± 10	.005*
LA active emptying fraction	48 ± 8	46 ± 8	50 ± 6	.066
LA strain parameters				
LA reservoir strain	24.8 ± 4.7	27.2 ± 3.3	20.2 ± 3.5	<.001*
LA conduit strain	−13.4 ± 5.0	−14.5 ± 4.0	−12.3 ± 5.2	.004*
LA contractile strain	−11.4 ± 4.8	−13.2 ± 3.7	−8.0 ± 4.2	<.001*
LV structure and function				
LV maximal volume, ml/m^2^	52.5 ± 13.9	51.8 ± 12.5	53.7 ± 18.3	.578
LV ejection fraction, %	59 ± 6	61 ± 5	57 ± 7	.016*
LV global longitudinal strain, %	−19.9 ± 3.2	−21.2 ± 2.7	−17.4 ± 2.5	<.001*

Abbreviations: MACEs, major adverse cardiovascular events; MHD, maintenance hemodialysis; 3DSTE, three‐dimensional speckle tracking echocardiography.

* *p*<.05 compared between groups with and without MACEs.

### 3.4 Predictors of MACEs

Univariate Cox proportional hazard model showed variates included age, history of diabetes, cTnT, LVMI, E/e', LVGLS, LAVi, LAPEF, LASr, LAScd significantly associated with MACEs (Table 2). A series of multivariate Cox models were then constructed to adjust confounding risk factors (Models 1–4, Table 3). As there was significant collinearity between LASr and LVGLS (Table S4), two nested models (Models 2 and 3) including LASr and LVGLS separately were built and compared and the *χ* ^2^ was 38.35 in Model 2 and 24.16 in Model 3. Therefore, we included age, cTnT, LVMI, LAVi, and LASr in Model 4. And LASr and LAVi remained significant (hazard ratio [HR], 0.69; 95% confidence interval [95% CI], 0.54–0.89, *p *= .004 for LASr; HR, 1.36; 95% CI, 1.03–1.35, *p *= .026 for LAVi).

**Table 2:** Univariate Cox proportional hazard model of major adverse cardiovascular events during follow‐up in the MHD cohort

Variable	Unit of increase	Hazard ratio (95% confidence interval)	*p*‐value
Age	1 year	1.05 (1.02–1.08)	<.001
History of diabetes	Yes or no	1.64 (0.79–3.42)	.051
cTnT	1 pg/ml	1.02 (1.01–1.04)	.001
LV mass index	1 g/m^2^	1.02 (1.00–1.03)	<.001
E/e'	1	1.22 (1.09–1.36)	.001
LV ejection fraction	1%	0.96 (0.89–1.02)	.196
LV global longitudinal strain	1%	1.87 (1.48–2.22)	<.001
LA maximal volume	1 ml/m^2^	1.14 (1.07–1.23)	<.001
LA emptying fraction	1%	0.95 (0.90–1.00)	.185
LA expansion index	1%	0.39 (0.15–1.03)	.156
LA passive emptying fraction	1%	0.02 (0.00–1.12)	.057
LA active emptying fraction	1%	0.29 (0.00–21.82)	.574
LA reservoir strain	1%	0.76 (0.69–0.84)	<.001
LA conduit strain	1%	1.05 (0.99–1.11)	.080
LA contractile strain	1%	1.08 (1.02–1.13)	.109

Abbreviations: cTnT, cardiac troponin T; LA, left atrium; MHD, maintenance hemodialysis.

**Table 3:** Multivariate Cox proportional hazard model of major adverse cardiovascular events during follow‐up in the MHD cohort

Model	Hazard ratio	95% confidence interval	*χ* ^2^	*p*‐value
Nested model 1: clinical variables	‐	‐	20.46	<.001
Age, years	1.05	1.02–1.09	‐	.002
History of diabetes, yes or no	0.75	0.28–1.99	‐	.565
cTnT, pg/ml	1.01	1.00–1.03	‐	.061
Nested model 2: echocardiographic variables (including LASr)	‐	‐	38.35	<.001
LVMI, g/m^2^	1.05	1.00–1.11	‐	.025
LASr, %	0.75	0.60–0.94	‐	.002
LA maximal volume, L/m^2^	1.27	1.09–1.28	‐	.013
E/e'	0.93	0.67–1.31	‐	.216
Nested model 3: echocardiographicVariables (including LVGLS)	‐	‐	24.16	<.001
LVMI, g/m^2^	1.05	1.00–1.10	‐	.031
LV global longitudinal strain, %	1.38	1.01–1.88	‐	.009
LA maximal volume, L/m^2^	1.11	0.99–1.24	‐	.018
E/e'	0.84	0.61–1.16	‐	.290
Nested model 4: clinical and echocardiographic variables	‐	‐	35.66	<.001
Age, years	1.10	0.99–1.22	‐	.082
cTnT, pg/ml	1.00	0.94–1.05	‐	.722
LVMI, g/m^2^	1.10	1.00–1.21	‐	.051
LA maximal volume, L/m^2^	1.36	1.03–1.35	‐	.026
LASr, %	0.69	0.54–0.89	‐	.004

Abbreviations: cTnT, cardiac troponin T; LA, left atrium; LASr, LA reservoir strain; LVGLS, LV global longitudinal strain; MHD, maintenance hemodialysis.

### 3.5 LASr as a predictor of MACEs

**Figure 1:**
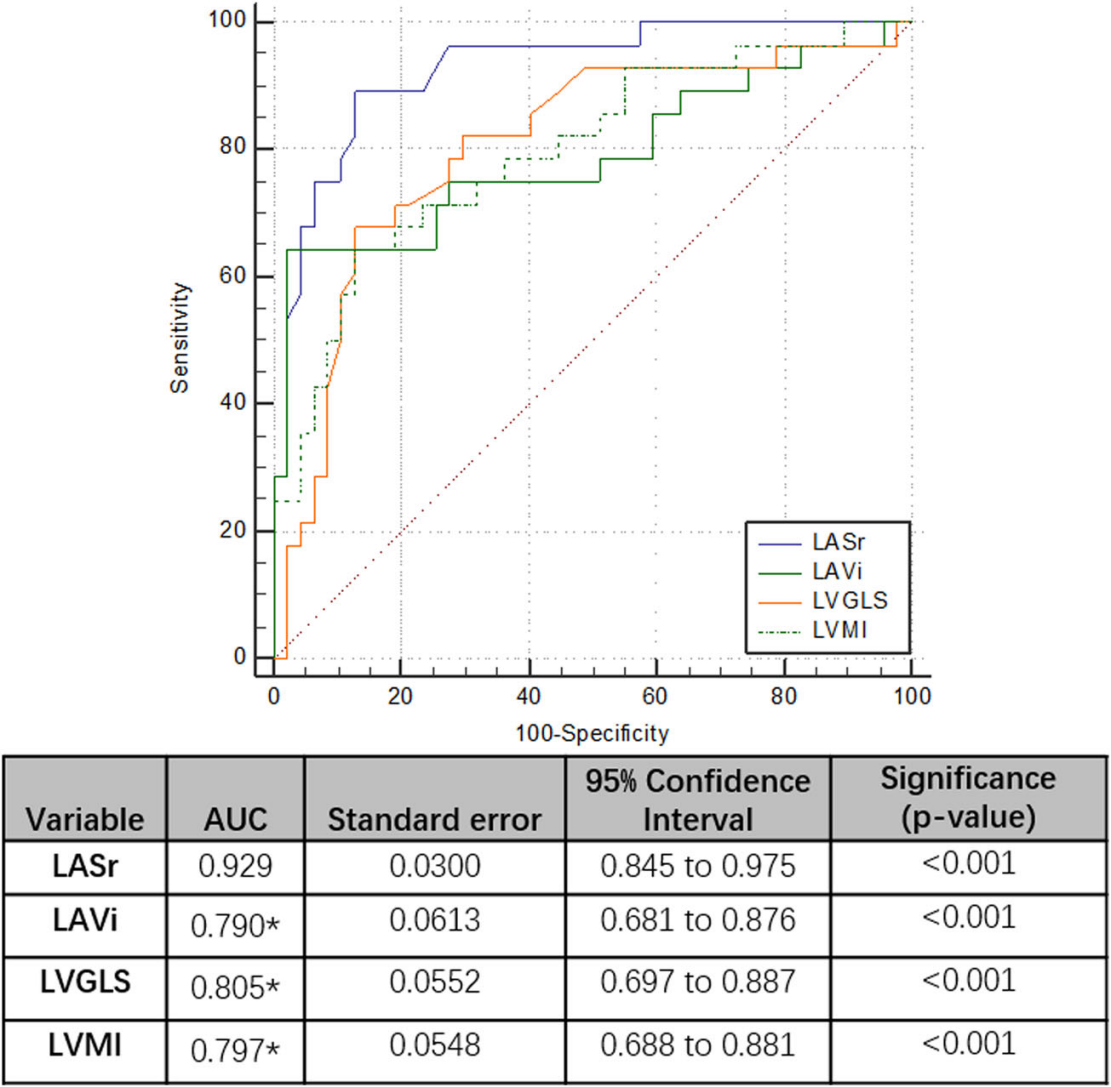
Receiver operating characteristic (ROC) curves of left atrium reservoir strain (LASr) compared to LV global longitudinal strain (LVGLS), LVMI, and LAVi in predicting all‐cause death and major adverse cardiovascular event. *p < .05 compared with ROC of LASr on DeLong test. The optimal threshold of LASr for the prediction of cardiovascular events was less than 24.2%, with 88.4% sensitivity and 84.0% specificity. AUC, area under curve

To evaluate the clinical utility of LASr as a biomarker of MACEs, we built the ROC curve of LASr and compared it to those of LVGLS, LAVi, and LVMI for MACEs, which were all traditional predictors of MACEs (Figure 1). Among all the echocardiographic indices, LASr was the strongest predictors of MACEs (AUC: 0.929, 95% CI: 0.845–0.975, *p* < .001, with the optimal threshold more than 24.2%, with 88.4% sensitivity and 84.0% specificity). DeLong tests showed that the AUC for LASr was significantly higher than those for LV GLS, LAVi, and LVMI.

**Figure 2:**
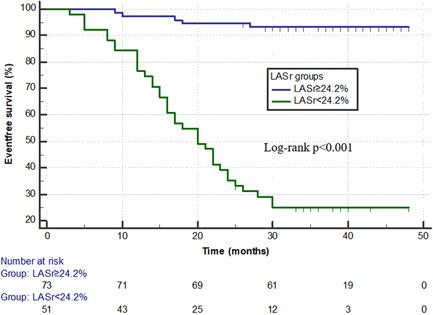
The Kaplan–Meier curves of major adverse cardiovascular events during follow‐up in maintenance hemodialysis cohort by normal and impaired left atrium reservoir strain (LASr) groups

To further determine the effect of impaired LASr on survival, we dichotomized LASr as normal (≥24.2%) or impaired (<24.2%) based on the optimal cutoff. Kaplan–Meier survival curves indicated incidence of MACEs in the impaired LASr group was significantly higher than that in the normal LASr group (log‐rank *p* < .001) (Figure 2).

### 3.6 Factors related to LASr

Univariate associations with LASr are summarized in Table S4. LASr was positively correlated with age, SBP, hypercholesterolemia, blood concentration of NT‐proBNP, cTnt and SCr, LVMI, and LVGLS (all *p* < .1). Stepwise regression showed LASr was independently correlated with age, SBP, and LVGLS among these demographic and biochemical data we collected (Table S4).

### 3.7 Reproducibility

Intra‐observer measurements showed ICC = 0.99 (95% CI: 0.97–0.99, *p* < .001) for LASr, 0.96 (95% CI: 0.92–0.98, *p* < .001) for LAScd, 0.94 (95% CI: 0.90–0.98, *p* < .001) for LASct, 0.95 (95% CI: 0.87–0.98, *p *< .001) for LAV‐max, 0.90 (95% CI: 0.85–0.97, *p *< .001) for LAV‐min, 0.91 (95% CI: 0.86–0.98, *p *< .001) for LAV‐preA. Similarly, interobserver measurement showed ICC = 0.95 (95% CI: 0.80–0.98, *p* < .001) for LASr, 0.92 (95% CI: 0.79–0.96, *p *< .001) for LAScd, 0.91 (95% CI: 0.78–0.96, *p *< .001) for LASct, 0.92 (95% CI: 0.84–0.97, *p *< .001) for LAV‐max, 0.87 (95% CI: 0.78–0.94 *p *< .001) for LAV‐min, 0.88 (95% CI: 0.80–0.97, *p *< .001) for LAV‐preA, indicating satisfactory reproducibility of 3DSTE measurements (Figure S3).

## 4 DISCUSSION

Our data showed that in MHD patients with no evidence of previous cardiac diseases, more than one‐third (43/124) encountered adverse cardiovascular events or death during the follow‐up about 4 years. LA was more often enlarged and LA strains were reduced in patients who experienced MACEs. LASr showed the best predictive efficiency of MACEs among other classic echocardiographic predictors. Furthermore, LA strain indices, especially LASr, showed better stability to fluid fluctuation than volumetric indices, which was particularly meaningful for the evaluation of MHD patients.

Cardiovascular complications are the most common cause of death in patients undergoing MHD.^1^ In contrast to the assessment of LV function, there is a paucity of literature focusing on the evaluation of LA. However, a growing body of evidence had demonstrated LA structure and function were associated with adverse cardiovascular events in a wide spectrum of diseases.^4^, ^5^, ^6^, ^7^ Previous research usually utilized two‐dimensional (2D) and Doppler echocardiography to assess LA size and function. However, the problem of geometric assumption was the main drawback for 2D echocardiography as susceptibility to multiple conditions for doppler echocardiography. The newly developed 3DSTE, whose accuracy and reproducibility have been verified as comparable to CT and MRI, should be a more suitable application than conventional echocardiography for the comprehensive assessment of LA.^9^, ^14^ Moreover, LA volumes measured by 3DSTE have independent and incremental prognostic value over those derived from 2D echocardiography.^4^ However, as 3DSTE data are relatively deficient, it is heretofore difficult to extrapolate cut points of LA parameters derived from 3DSTE. To our knowledge, this study was the first to evaluate LA size and function via 3DSTE in MHD patients, along with enriching the database of clinical evidence‐based medicine.

### 4.1 LA size in MHD patients

Previous research showed that MHD patients had a larger LA than normal subjects, which was detected both by 2D echocardiography and 3DSTE.^15^, ^16^, ^17^ LA dilation and atrial fibrillation are common in MHD patients as a result of volume overload, high incidence of hypertension, and some endocrine changes.^18^ Moreover, body fluid fluctuation, electrolyte disorder, uremic toxins, LV diastolic dysfunction, and enhanced neurohormonal activation also accompany MHD patients and contribute to LA enlargement.^17^ Previous literature suggested LAVi as a crucial marker of adverse cardiovascular outcomes.^19^ The American Society of Echocardiography (ASE) considers LA enlargement as LAVi > 28 ml/m^2^ (derived from 2D echocardiography) for predicting cardiac events.^20^ In MHD cohort, Barberato^15^ previously reported 2D derived LAVi >35 ml/m^2^ as an independent predictor of intradialytic hypotension, an important complication of hemodialysis treatment which contributes to the development of heart failure and increased mortality. Our data also demonstrated that LAVi was an independent predictor of MACEs (Table 3, Figure 1). However, the volume indices are load‐dependency and in our study, LA size varied notably at different time points during hemodialysis cycle (Table S3). Considering the body fluid fluctuation in MHD patients and the load‐dependency of LA size,^21^ the time of image acquirements was quite crucial for LA assessment.

### 4.2 LA phasic function and LA strain in MHD patients

Nowadays, being recognized as sensitive markers of LV diastolic dysfunction,^4^ LA phasic functions have raised massive interests. Previous studies have suggested that LA functional changes precede transformation of LA size in LV diastolic dysfunction.^6^ During the cardiac cycle, the role of LA varies from a reservoir during LV systole, a conduit for blood transiting from the pulmonary veins to the LV during early diastole to an active booster that supplements LV ventricular filling in late diastole. LA phasic functions could be derived from strain and volumetric method. By means of 3DSTE, LA phasic volumes and strain parameters could be acquired with higher accuracy and convenience than by 2D echocardiography. Our study demonstrated LA reservoir, conduit, and contractile strains were all impaired in the cohort with MACEs. LA conduit function is more reliant on LV diastolic function, including both the suction force hinging on LV relaxation and LV stiffness, whereas LA booster function is more based on intrinsic LA contractility. LASr is the sum of the absolute values of LAScd and LASct. Thus it is an index that can comprehensively reflect the LV diastolic function and the left atrial active systolic function.

LAVi is a surrogate indicator of LV diastolic dysfunction. However, it is an insensitive biomarker in early stage of disease progression.^22^ As the primary function of the LA is to modulate LV filling, it is reasonable that LA phasic function changes at the earliest stage. Between the volumetric and strain methods, LA phasic strains were recommended over LA volume parameters for a less degree of preload dependency.^23^ Among the three LA strain metrics, LASr has been reported to be a prognostic biomarker across a spectrum of acute and chronic cardiovascular pathologies.^24^ Our study also showed the outstanding predictive value of LASr for MACEs in MHD patients. In addition to age and gender, LA strain parameters were reported to be independently associated with LV systolic and diastolic function metrics.^25^ Consistent with previous studies, our study showed LASr was negatively correlated with age, SBP, and LVGLS. Additionally, LASr was also negatively correlated with LVMI and the plasma concentration of cTnT, NT‐proBNP, and SCr, all of which were adverse prognostic factors in MHD patients,^26^, ^27^, ^28^ indicating that LASr is related to the overall condition, especially cardiac performance of patients. Therefore, we propose that LA strain indices should be incorporated in clinical evaluation and follow‐up of MHD patient.

### 4.3 Impaired LA strain and arrhythmias

In our cohort with severe arrhythmias excluded at enrollment, MACEs contained 23 (over 50% in 43) cases of severe arrhythmias, including 13 atrial fibrillations. The prevalence of arrhythmias is quite high in MHD patients.^29^ The mechanism behind the development of arrhythmias is complex and not fully elucidated. It involves the interaction of several various factors, including older age, structural changes of the heart (LA enlargement, LV hypertrophy, and dilatation),^30^ hypertension, volume overload, electrolyte abnormalities, dialysis‐induced myocardial ischemia, and so on.^31^, ^32^ In other disease spectrums, impaired LASr predicts atrial fibrillation recurrence following catheter ablation^33^ and helps the diagnosis of HF with preserved ejection fraction.^34^ Based on our data and previous literature, we could speculate there also exists a link between arrhythmias, especially atrial fibrillation, and impaired LA strain in MHD cohort.

### 4.4 Limitations

Since LA strain is not completely preload independent, it is important to select the evaluation time point. Here we recommended with cautions LA evaluation should be conducted on interdialytic day in MHD cohorts, just as recommended for LV assessment by previous literature.^17^, ^35^ Secondly, LA appendage was not included for the calculation of LA volume and function. As LA appendage was reported to play an important role in the LA volume measurement,^36^ we are looking forward to new analysis software incorporating LA appendage to obtain more accurate data.

## 5 CONCLUSIONS

In summary, our study assessed LA volumes and phasic function via 3DSTE in MHD patients and investigated predictors for MACEs. The results presented LASr derived from 3DSTE was an important predictor of MACEs in MHD patients, superior to LAVi, clinical, and LV parameters including LVMI and LVGLS. The finding remained after adjustment for conventional and unconventional risk factors. Further study is needed to elucidate the underlying mechanism. LA strain assessment contributes to cardiovascular risk stratification in MHD cohorts.

## CONFLICTS OF INTEREST

The authors declare no conflicts of interest.

## Supporting information

Figure S1: Demonstration of three‐dimensional speckle tracking echocardiography (3DSTE) report for LA in Tomtec station: a normal control (the former) and a MHD patient.

Figure S2: Changes of left atrial parameters derived from 3DSTE on interdialytic days, pre and post hemodialysis.

Figure S3: Bland‐Altman analysis for intra‐(circle) and inter‐(triangle) observer reliability of LA parameters derived from 3DSTE.

Supporting information.

## Data Availability

The data that support the findings of this study are available from the corresponding author upon reasonable request.
