# Supplementary figures and images for: Left atrial reservoir strain is an outstanding predictor of adverse cardiovascular outcomes in patients undergoing maintenance hemodialysis: Assessment via three‐dimensional speckle tracking echocardiography
Source: Clin Cardiol. 2022 Mar 21;45(5):549–57. doi: 10.1002/clc.23815 (PMC9045074; doi:10.1002/clc.23815)

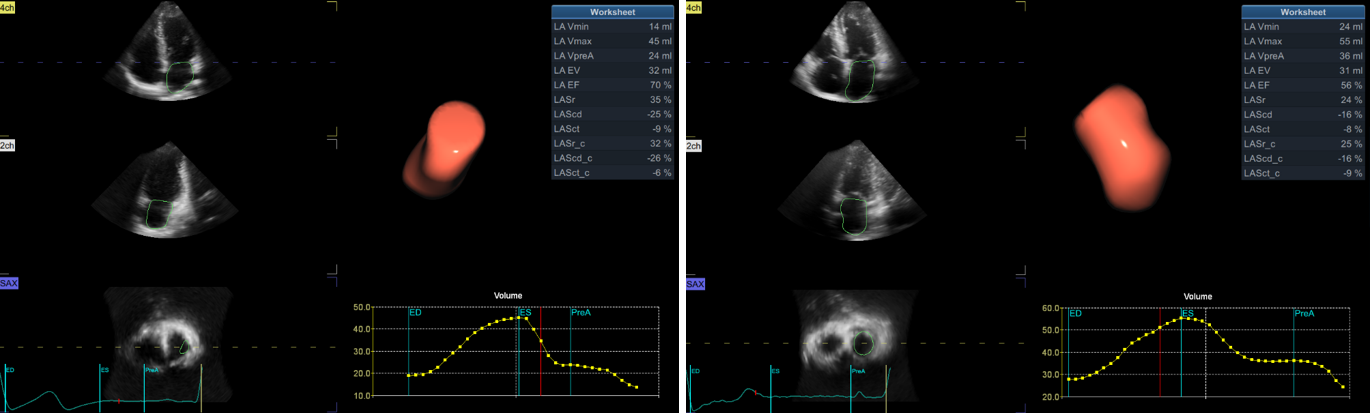

Supplement: Supplementary file 1 — Figure S1: Demonstration of three‐dimensional speckle tracking echocardiography (3DSTE) report for LA in Tomtec station: a normal control (the former) and a MHD patient. [file CLC-45-549-s003.tif]

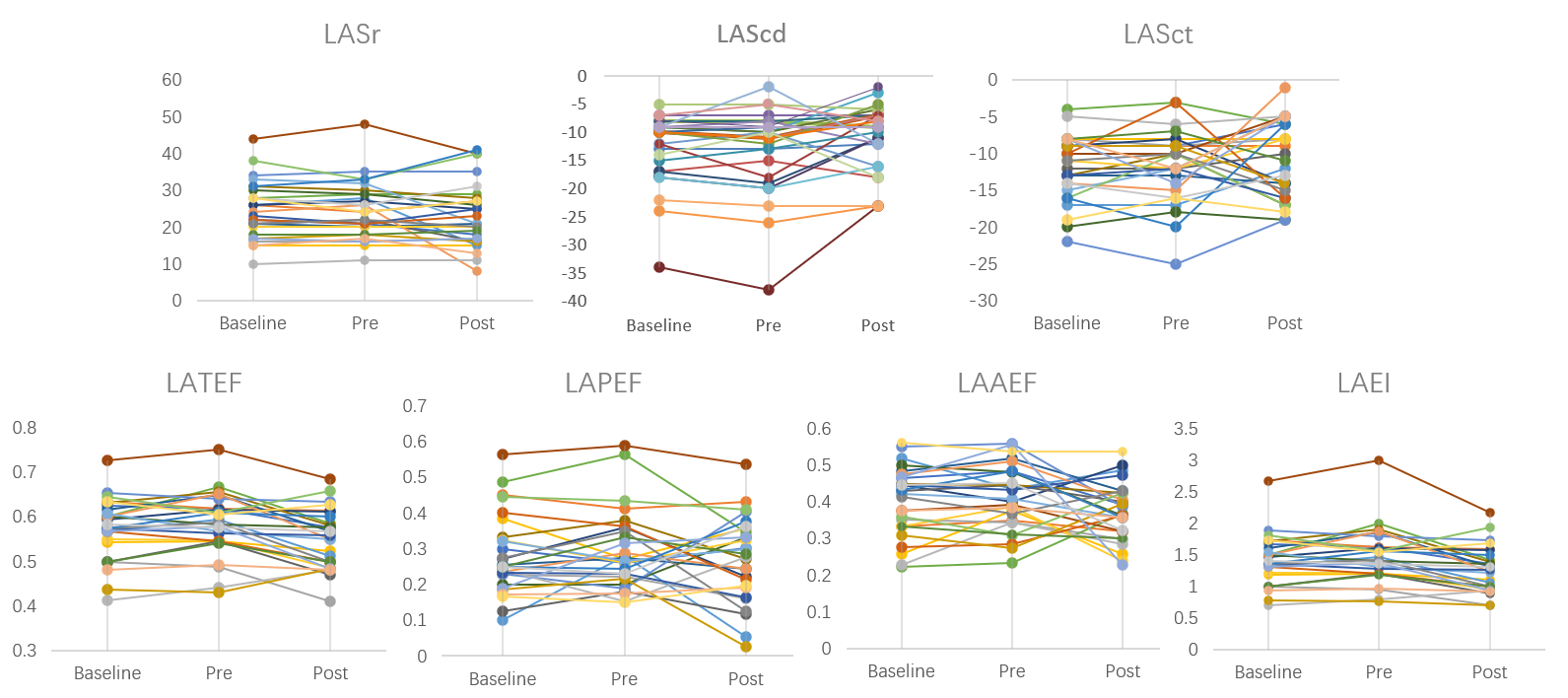

Supplement: Supplementary file 2 — Figure S2: Changes of left atrial parameters derived from 3DSTE on interdialytic days, pre and post hemodialysis. [file CLC-45-549-s001.tif]

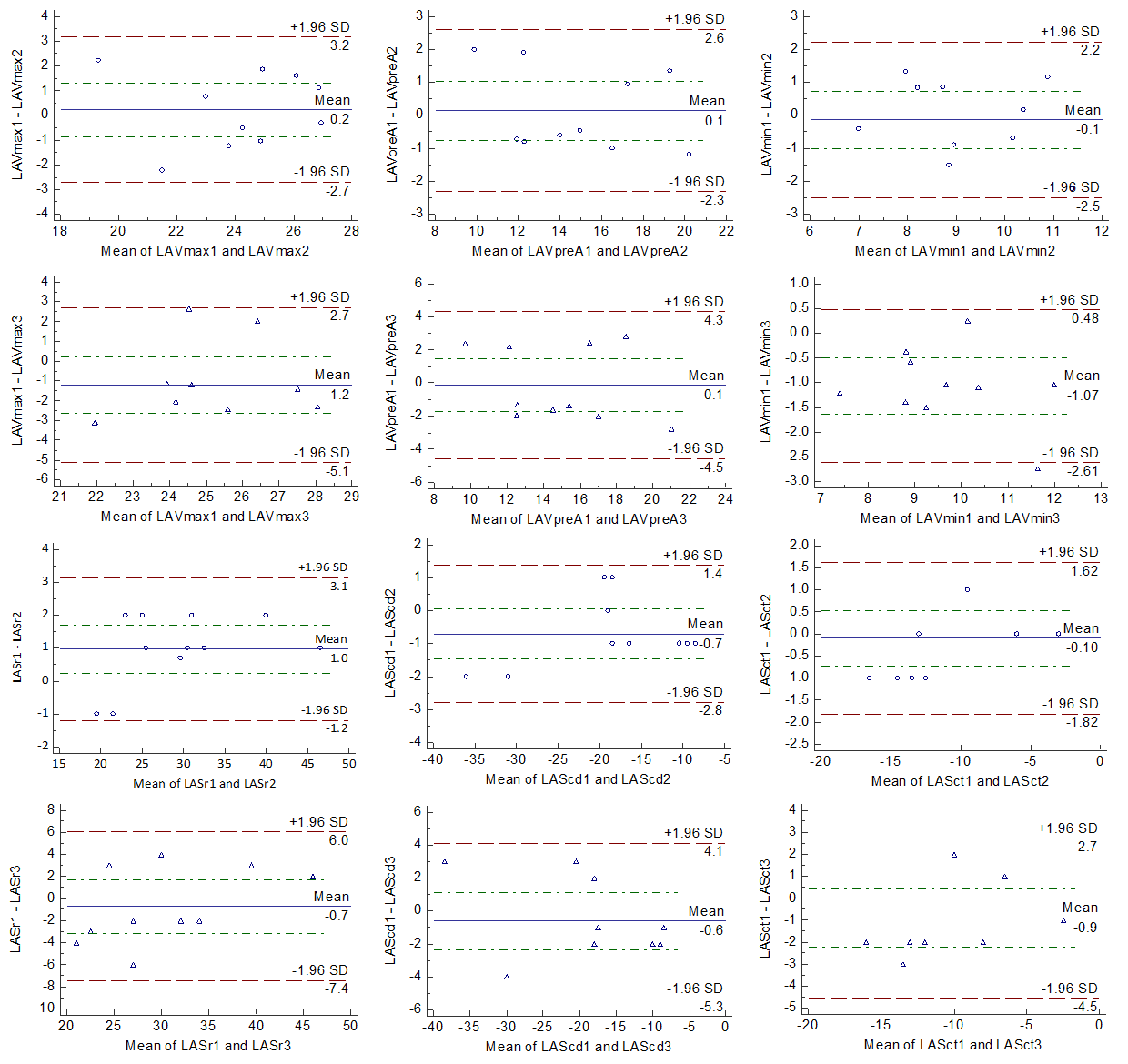

Supplement: Supplementary file 3 — Figure S3: Bland‐Altman analysis for intra‐(circle) and inter‐(triangle) observer reliability of LA parameters derived from 3DSTE. [file CLC-45-549-s002.tif]
