# Supplementary material for: Left atrial reservoir strain is an outstanding predictor of adverse cardiovascular outcomes in patients undergoing maintenance hemodialysis: Assessment via three‐dimensional speckle tracking echocardiography
Source: Clin Cardiol. 2022 Mar 21;45(5):549–57. doi: 10.1002/clc.23815 (PMC9045074; doi:10.1002/clc.23815)
Supplement: Supplementary file 4 — Supporting information. [file CLC-45-549-s004.docx]

**Table S1：Comparison of baseline demographic, clinical and biochemical characteristics of the MHD cohort grouped by MACEs**

|  | **MHD**  **n=124** | **Grouping by MACEs** | | |
| --- | --- | --- | --- | --- |
|  |  | **No (n=81)** | **Yes (n=43)** | ***P* value** |
| **Demographic characteristics** |  |  |  |  |
| **Age, y** | 57±12 | 56±13 | 62±13 | 0.010 |
| **Gender, male (%)** | 64(52%) | 42(52%) | 22(51%) | 1.000 |
| **Body mass index(kg/m^2^)** | 22.0±3.7 | 21.7±3.7 | 22.3±3.7 | 0.378 |
| **Systolic blood pressure, mmHg** | 148±20 | 150±26 | 146±22 | 0.342 |
| **Diastolic blood pressure, mmHg** | 83±13 | 84±14 | 81±12 | 0.264 |
| **Past history** |  |  |  |  |
| **Dialysis duration, year** | 5.3±4.5 | 5.7±5.0 | 4.5±3.4 | 0.224 |
| **Ultrafiltration volume, L/m^2^** | 1.3±0.4 | 1.4±0.5 | 1.3±0.4 | 0.754 |
| **Current smoking history** | 12(10%) | 8(10%) | 4(9%) | 1.000 |
| **History of diabetes, n (%)** | 19(15%) | 8(10%) | 11(26%) | 0.034 |
| **History of primary hypertension, n(%)** | 53(43%) | 33(41%) | 20(47%) | 0.571 |
| **Hypercholesterolemia, n(%)** | 32(26%) | 20(25%) | 12(28%) | 0.252 |
| **History of Coronary heart diseases, n(%)** | 15(12%) | 9(11%) | 6(14%) | 0.773 |
| **Biochemical Characteristics** |  |  |  |  |
| **cTnT, pg/ml** | 11.5(3.8, 28.8) | 7.7(3.8, 19.5) | 12.1(3.5, 31.5) | 0.063 |
| **Log (BNP), log(pg/ml)** | 3.6±0.4 | 3.5±0.5 | 3.6±0.4 | 0.304 |
| **BUN, mmol/L** | 28.3±5.6 | 28.2±5.6 | 28.5±5.6 | 0.850 |
| **SCr, μmol/L** | 1117±262 | 1136±278 | 1082±230 | 0.440 |
| **Uric acid, μmol/L** | 453±75 | 452±78 | 457±71 | 0.834 |
| **Albumin, g/L** | 39.1±2.8 | 39.4±3.0 | 38.5±2.6 | 0.234 |
| **Cholesterol, mmol/L** | 4.3±0.9 | 4.3±1.0 | 4.3±0.8 | 0.882 |
| **Triglyceride, mmol/L** | 1.6±1.2 | 1.6±1.3 | 1.5±1.0 | 0.722 |
| **Hemoglobin, g/L** | 112±16 | 110±18 | 114±13 | 0.457 |
| **Fasting blood-glucose, mmol/L** | 6.7±2.5 | 6.8±2.4 | 6.7±2.5 | 0.865 |
| **Calcium-phosphorus product,** **1mmol^2^/L^2^** | 4.9±1.3 | 5.1±1.5 | 4.9±1.3 | 0.690 |
| **Medications** |  |  |  |  |
| **α/β- Blocker, n (%)** | 62(50%) | 46(57%) | 16(37%) | 0.413 |
| **ACEI/ARB, n (%)** | 64(51%) | 44(54%) | 20(47%) | 0.453 |
| **CCB, n (%)** | 75(60%) | 52(64%) | 23(53%) | 0.255 |

Values are presented as means ± SD or medians (interquartile ranges) for continuous varibles and number (%) for categorical variables.

MHD: maintenance hemodialysis; MACEs: major adverse cardiovascular events; BUN: Blood Ureanitrogen; SCr: Serum creatinine.

**Table S2: Comparison of conventional echocardiography parameters in MHD cohort grouped by MACEs**

|  | **MHD**  **n=124** | **Grouping by cardiovascular events** | | |
| --- | --- | --- | --- | --- |
|  |  | **No (n=81)** | **Yes (n=43)** | ***P* value** |
| **Heart rate, min^-1^** | 77±11 | 76±11 | 78±12 | 0.316 |
| **LV mass index, g/m^2^** | 135.3±35.8 | 121.1±24.1 | 158.0±39.8 | <0.001 |
| **LA diameter^△^, mm** | 40.1±5.0 | 39.3±5.0 | 41.3±4.9 | 0.103 |
| **E, cm/s** | 73.4±17.0 | 72.5±18.3 | 74.4±24.9 | 0.706 |
| **A, cm/s** | 96.9±20.9 | 93.7±19.9 | 102.1±21.8 | 0.087 |
| **E/A** | 0.8±0.3 | 0.8±0.2 | 0.7±0.3 | 0.262 |
| **DT, ms** | 167±40 | 168±38 | 166±44 | 0.830 |
| **IVRT, ms** | 106±28 | 102±26 | 112±30 | 0.123 |
| **IVCT, ms** | 72±19 | 71±20 | 73±17 | 0.689 |
| **PASP, mmHg** | 35±8 | 34±8 | 37±8 | 0.177 |
| **S-sep, cm/s** | 8.4±1.4 | 8.6±1.5 | 7.9±1.3 | 0.025 |
| **e’-sep, cm/s** | 7.5±2.2 | 7.9±2.2 | 6.9±2.1 | 0.051 |
| **a’-sep, cm/s** | 10.7±2.3 | 10.9±2.2 | 10.4±2.4 | 0.399 |
| **e’/a’-sep** | 0.7±0.2 | 0.7±0.2 | 0.7±0.2 | 0.246 |
| **S-lat, cm/s** | 9.8±1.8 | 9.9±1.8 | 9.6±1.8 | 0.503 |
| **e’-lat, cm/s** | 9.5±2.5 | 10.0±2.7 | 8.7±2.1 | 0.029 |
| **a’-lat, cm/s** | 12.5±2.7 | 12.3±2.5 | 12.9±3.0 | 0.334 |
| **e’/a’-lat** | 0.8±0.3 | 0.9±0.3 | 0.7±0.3 | 0.027 |
| **E/e’** | 8.9±3.0 | 8.3±2.2 | 9.5±4.1 | 0.012 |

^△^：LA diameter was measured from parasternal long-axis view. MHD: maintenance hemodialysis; MACEs: major adverse cardiovascular events.

E, A, E/A:left ventricular early (E), late (A) inflow velocities, ratio between E and A velocities (E/A ratio) measured by pulsed Doppler placing the sample volume in between the tips of the mitral valve; PASP: pulmonary artery systolic pressure;

S-sep, e’-sep, a’-sep, S-lat, e’-lat, a’-lat: myocardial velocities in peak systole (S-lat, S-sep), early (e'-lat, e’-sep) and late diastole (a'-lat, a’-sep) measured by Pulsed-wave tissue Doppler imaging (TDI) of the mitral annulus when the sample volume was placed in septal and lateral annulus of the wall; IVRT, IVCT: isovolumic relaxation/contraction time acquired from TDI of the septal and lateral annulus;

E/e’: ratio between E wave and the average of e’-sep and e’-lat.

**Table 1：Comparison of 3DSTE parameters in MHD cohort grouped by MACEs**

|  | **MHD**  **n=124** | **Grouping by MACEs** | | |
| --- | --- | --- | --- | --- |
|  |  | **No (n=81)** | **Yes (n=43)** | ***P* value** |
| **LA volume parameters, ml/m^2^** |  |  |  |  |
| **LA maximal volume** | 38.0±10.5 | 33.8±6.9 | 45.1±11.9 | <0.001† |
| **LA minimal volume** | 15.2±4.7 | 14.7±4.9 | 16.0±4.5 | 0.227 |
| **LA pre-systolic volume** | 29.3±4.7 | 27.6±8.9 | 32.1±9.1 | 0.038† |
| **LA functional parameters, %** |  |  |  |  |
| **LA total emptying fraction** | 60±5 | 61±4 | 59±5 | 0.237 |
| **LA expansion index** | 155±29 | 158±28 | 150±31 | 0.285 |
| **LA passive emptying fraction** | 23±10 | 26±10 | 19±10 | 0.005† |
| **LA active emptying fraction** | 48±8 | 46±8 | 50±6 | 0.066 |
| **LA strain parameters** |  |  |  |  |
| **LA reservoir strain** | 24.8±4.7 | 27.2±3.3 | 20.2±3.5 | <0.001† |
| **LA conduit strain** | -13.4±5.0 | -14.5±4.0 | -12.3±5.2 | 0.004† |
| **LA contractile strain** | -11.4±4.8 | -13.2±3.7 | -8.0±4.2 | <0.001† |
| **LV structure and function** |  |  |  |  |
| **LV maximal volume, ml/m^2^** | 52.5±13.9 | 51.8±12.5 | 53.7±18.3 | 0.578 |
| **LV ejection fraction, %** | 59±6 | 61±5 | 57±7 | 0.016† |
| **LV global longitudinal strain, %** | -19.9±3.2 | -21.2±2.7 | -17.4±2.5 | <0.001† |

†:P<0.05 compared between groups with and without MACEs. MHD: maintenance hemodialysis; MACEs: major adverse cardiovascular events.

**Table S3：** **Changes of left atrial parameters derived from 3DSTE on interdialytic days, pre and post hemodialysis**

|  |  | **Interdialytic** | **Pre** | **Post** | ***P*_1_** | ***P*_2_** | ***P*_3_** |
| --- | --- | --- | --- | --- | --- | --- | --- |
| **Volume parameters** | **LAVmax** | 40.9±10.7 | 43.2±13.3 | 35.9±11.8 | 0.000 | 0.001 | 0.000 |
|  | **LAVpreA** | 29.3±9.2 | 30.8±12.5 | 25.9±9.4 | 0.007 | 0.007 | 0.002 |
|  | **LAVmin** | 16.8±5.7 | 17.6±7.3 | 15.9±5.9 | 0.001 | 0.124 | 0.012 |
| **Global/reservoir function** | **LATEF** | 57.8±7.1 | 58.3±6.8 | 54.9±6.5 | 0.142 | 0.001 | 0.000 |
|  | **LAEI** | 145±51 | 146±44 | 126±36 | 0.142 | 0.000 | 0.000 |
|  | **LASr** | 24.5±7.6 | 24.4±7.7 | 23.0±8.7 | 0.769 | 0.133 | 0.222 |
| **Conduit** | **LAPEF** | 28.1±11.1 | 29.3±11.0 | 27.2±11.8 | 0.357 | 0.594 | 0.359 |
|  | **LAScd** | -12.6±6.3 | -12.9±7.5 | -10.9±7.5 | 0.646 | 0.017 | 0.076 |
| **Booster pump** | **LAAEF** | 40.3±9.5 | 41.1±8.7 | 37.2±7.7 | 0.331 | 0.066 | 0.060 |
|  | **LASct** | -11.8±4.4 | -11.6±4.9 | -10.8±5.2 | 0.684 | 0.249 | 0.497 |

*P*_1_: Interdialytic versus Pre; *P*_2_: Interdialytic versus Post; *P*_3_: Pre versus Post

**Table 2: Univariate Cox proportional hazard model of first cardiovascular event during follow-up in the MHD cohort**

| **Variable** | **Unit of increase** | **Hazard Ratio**  **(95% Confidence Interval)** | **P Value** |
| --- | --- | --- | --- |
| **Age** | 1yr | 1.05(1.02~1.08) | <0.001 |
| **History of diabetes** | Yes or no | 1.64(0.79~3.42) | 0.051 |
| **cTnT** | 1pg/ml | 1.02(1.01~1.04) | 0.001 |
| **LV mass index** | 1 g/m^2^ | 1.02(1.00~1.03) | <0.001 |
| **E/e’** | 1 | 1.22(1.09~1.36) | 0.001 |
| **LV ejection fraction** | 1% | 0.96(0.89~1.02) | 0.196 |
| **LV global longitudinal strain** | 1% | 1.87(1.48~2.22) | <0.001 |
| **LA maximal volume** | 1ml/m^2^ | 1.14(1.07~1.23) | <0.001 |
| **LA emptying fraction** | 1% | 0.95(0.90~1.00) | 0.185 |
| **LA expansion index** | 1% | 0.39(0.15~1.03) | 0.156 |
| **LA passive emptying fraction** | 1% | 0.02(0.00~1.12) | 0.057 |
| **LA active emptying fraction** | 1% | 0.29(0.00~21.82) | 0.574 |
| **LA reservoir strain** | 1% | 0.76(0.69~0.84) | <0.001 |
| **LA conduit strain** | 1% | 1.05(0.99~1.11) | 0.080 |
| **LA contractile strain** | 1% | 1.08(1.02~1.13) | 0.109 |

**Table 3:** **Multivariate Cox proportional hazard model of first cardiovascular event during follow-up in the MHD cohort**

| **Model** | **Hazard Ratio** | **95% confidence interval** | **χ^2^** | **P Value** |
| --- | --- | --- | --- | --- |
| **Nested model 1: clinical variables** |  |  | 20.46 | <0.001 |
| **Age, years** | 1.05 | 1.02~1.09 |  | 0.002 |
| **History of diabetes, Yes or no** | 0.75 | 0.28~1.99 |  | 0.565 |
| **cTnT, pg/ml** | 1.01 | 1.00~1.03 |  | 0.061 |
| **Nested model 2: echocardiographic**  **Variables (including LASr)** |  |  | 38.35 | <0.001 |
| **LVMI, g/m^2^** | 1.05 | 1.00~1.11 |  | 0.025 |
| **LA reservoir strain, %** | 0.75 | 0.60~0.94 |  | 0.002 |
| **LA maximal volume, L/m^2^** | 1.27 | 1.09~1.28 |  | 0.013 |
| **E/e’** | 0.93 | 0.67~1.31 |  | 0.216 |
| **Nested model 3: echocardiographic**  **Variables (including LVGLS)** |  |  | 24.16 | <0.001 |
| **LVMI, g/m^2^** | 1.05 | 1.00~1.10 |  | 0.031 |
| **LV global longitudinal strain, %** | 1.38 | 1.01~1.88 |  | 0.009 |
| **LA maximal volume, L/m^2^** | 1.11 | 0.99~1.24 |  | 0.018 |
| **E/e’** | 0.84 | 0.61~1.16 |  | 0.290 |
| **Nested model 4: clinical and echocardiographic variables** |  |  | 35.66 | <0.001 |
| **Age, years** | 1.10 | 0.99~1.22 |  | 0.082 |
| **cTnT, pg/ml** | 1.00 | 0.94~1.05 |  | 0.722 |
| **LVMI, g/m^2^** | 1.10 | 1.00~1.21 |  | 0.051 |
| **LA maximal volume, L/m^2^** | 1.36 | 1.03~1.35 |  | 0.026 |
| **LA reservoir strain, %** | 0.69 | 0.54~0.89 |  | 0.004 |

**Table S4** **Univariate and** **multivariate regression analysis for predictors of LASr**

|  | **Univariate** | |  | **Model：demographic+biochemical** | |  | |
| --- | --- | --- | --- | --- | --- | --- | --- |
|  | **Coefficient** | ***P*** | | **β Coefficient(95%CI)** | ***P*** | |  |
| **Age, y** | -0.17 | 0.008 |  | -0.32(-0.50~-0.13) | 0.001 |  |  |
| **Gender, male (%)** | 1.75 | 0.312 |  |  |  |  |  |
| **Body mass index(kg/m^2^)** | -0.27 | 0.240 |  |  |  |  |  |
| **Ultrafiltration volume, L/m^2^** | -0.02 | 0.702 |  |  |  |  |  |
| **Dialysis duration, year** | 0.14 | 0.394 |  |  |  |  |  |
| **Systolic blood pressure, mmHg** | -0.07 | 0.026 |  | 0.10(0.004~0.19) | 0.042 |  |  |
| **Diastolic blood pressure, mmHg** | 0.10 | 0.129 |  |  |  |  |  |
| **History of diabetes** | -4.06 | 0.305 |  |  |  |  |  |
| **History of primary hypertension** | 4.33 | 0.311 |  |  |  |  |  |
| **Hypercholesterolemia** | 4.01 | 0.091 |  | 3.59(-2.53~9.71) | 0.244 |  |  |
| **History of Coronary heart diseases** | -4.44 | 0.107 |  |  |  |  |  |
| **Current smoking** | 2.74 | 0.462 |  |  |  |  |  |
| **cTnT, pg/ml** | -0.11 | 0.078 |  | -0.03(-0.13~0.08) | 0.608 |  |  |
| **Log (BNP), log(pg/ml)** | -6.67 | 0.005 |  | -2.74(-8.06~2.57) | 0.304 |  |  |
| **BUN, mmol/L** | -0.04 | 0.851 |  |  |  |  |  |
| **SCr, μmol/L** | 0.02 | 0.016 |  | -0.01(-0.01~0.004) | 0.239 |  |  |
| **Uric acid, μmol/L** | -0.00 | 0.914 |  |  |  |  |  |
| **Albumin, g/L** | 0.52 | 0.176 |  |  |  |  |  |
| **Cholesterol, mmol/L** | -0.08 | 0.214 |  |  |  |  |  |
| **Triglyceride, mmol/L** | -0.13 | 0.486 |  |  |  |  |  |
| **Hemoglobin, g/L** | -0.02 | 0.827 |  |  |  |  |  |
| **Calcium-phosphorus product,** **1mmol^2^/L^2^** | 0.69 | 0.519 |  |  |  |  |  |
| **Fasting blood-glucose, mmol/L** | -0.47 | 0.303 |  |  |  |  |  |
| **LVMI, g/m^2^** | -0.11 | <0.001 |  | -0.01(-0.09~0.07) | 0.819 |  |  |
| **LVGLS, %** | -1.67 | <0.001 |  | -0.85(-1.56~-0.14) | 0.020 |  |  |
